# Impacts of leukocyte telomere length on incidence and severity of age-related cataract: a cross-cohort analysis

**DOI:** 10.1186/s40662-025-00465-x

**Published:** 2025-12-01

**Authors:** Xianqi Zheng, Ting Su, Chenxiao Shen, Guanrong Wu, Zijing Du, Xingchen Geng, Yuling Xu, Ningni Jiang, Qinyi Li, Jiahui Cao, Ying Fang, Yijun Hu, Mingguang He, Zhuoting Zhu, Xiayin Zhang, Honghua Yu

**Affiliations:** 1 Guangdong Eye Institute, Department of Ophthalmology, Guangdong Provincial People’s Hospital, Guangdong Academy of Medical Sciences, Southern Medical University, No. 106, Zhongshan Second Road, Guangzhou, China; 2 Guangzhou Aier Eye Hospital, Jinan University, Guangzhou, China; 3 School of Optometry, The Hong Kong Polytechnic University, Hong Kong, China; 4 Centre for Eye Research Australia, Ophthalmology, Department of Surgery, University of Melbourne, Melbourne, VIC Australia; 5 Singapore Eye Research Institute, Singapore National Eye Centre, Singapore, Singapore; 6 Guangdong Provincial Key Laboratory of Artificial Intelligence in Medical Image Analysis and Application, Guangdong Provincial People’s Hospital, Guangdong Academy of Medical Sciences, Guangzhou, China

**Keywords:** Leukocyte telomere length, Age-related cataract, Cross-cohort study, Phenome-wide association study

## Abstract

**Objective:**

To investigate the relationship between leukocyte telomere length (LTL), a biomarker of cellular aging, and both the incidence and severity of age-related cataract (ARC) across cohorts from the UK and China.

**Methods:**

The multicenter, multiethnic cohort study involved 122,932 healthy individuals with a mean age of 56.27 years from the UK Biobank, a community-based cohort, and 53 cataract patients with a mean age of 71.74 years from a hospital-based cohort in China. LTL was measured using validated polymerase chain reaction techniques. ARC was assessed using a combination of self-reported data, medical records, and operation codes. In the Chinese cohort, lens morphological features and opacities were evaluated using Scheimpflug imaging. Associations between LTL and ARC were analyzed using Cox proportional hazards models, logistic regression, and restricted cubic splines. A phenome-wide association study (PheWAS) was conducted to validate the association between LTL and cataract in the UK Biobank cohort.

**Results:**

Over a median follow-up time of 11.18 years, 4,089 incident ARC cases were documented in the UK cohort. Longer LTL was associated with a lower incidence of ARC [hazard ratio (HR) = 0.93, 95% confidence interval (CI): 0.91 to 0.96; *P* < 0.001]. Restricted cubic splines indicated an L-shaped association between LTL and ARC (*P* for nonlinearity = 0.03), where ARC risk decreased with longer LTL until a threshold before plateauing. The PheWAS provided support for the association between LTL and cataract (*P* = 2.36 × 10⁻⁶) across 1,011 phecodes in the UK Biobank. In the Chinese cohort, LTL was negatively correlated with average lens density (β = − 0.32, 95% CI: − 0.61 to − 0.04; *P* = 0.03).

**Conclusions:**

Longer LTL is associated with a reduced risk and severity of ARC, suggesting shared biological pathways between telomere attrition and lens aging. This supports the lens as a unique window for studying systemic aging and LTL as an index of modifiable health behaviors influencing cataract development.

**Supplementary Information:**

The online version contains supplementary material available at 10.1186/s40662-025-00465-x.

## Background

Cataracts remain the leading cause of global blindness among adults aged 50 years and older, affecting 15.2 million people worldwide in 2020 [1, 2]. Despite advancements in surgical techniques enabling effective cataract surgery with rapid visual recovery, cataract continues to pose a significant public health burden, particularly in aging populations with extended life expectancies [2–6].

As an age-associated condition, cataracts are often considered an inevitable aspect of aging [7]. However, individuals of the same chronological age can exhibit varying onset and severity of cataracts. Telomeres, consisting of nucleoprotein structures with tandem TTAGGG DNA sequences located at the ends of linear chromosomes, are recognized as in vivo biomarkers of biological aging [8–12]. Leukocyte telomere length (LTL) provides a practical surrogate for systemic telomere length due to its accessibility. Numerous studies have demonstrated that LTL shortening is associated with organismal aging and age-related pathologies including cardiovascular diseases, diabetes mellitus, and cancers [13–15]. Thus, investigating the associations between LTL and cataract may reveal mechanisms beyond chronological aging that drive cataractogenesis.

The hypothesis that lens transparency may be associated with LTL is supported by observations in individuals with Werner syndrome, characterized by accelerated telomere attrition and early onset of bilateral cataracts [16, 17]. Notably, telomere shortening in Werner syndrome stems from null mutations of the WRN gene, distinct from physiological telomere erosion [18]. Animal studies reported conflicting telomere-cataract associations. In brown Norway rats, lens epithelium telomere length (LETL), which was measured by fluorescence in situ hybridization (FISH), shortened with aging and correlated with reduced lens epithelial cell (LEC) replication rates, decreased clonal proliferative potential in vitro, and age-related cataract (ARC) formation [19]. Conversely, Southern blot analysis revealed longer LETL in cataractous dogs versus controls [20].

In 2,750 American community-dwelling older adults, LTL measured by quantitative polymerase chain reaction (qPCR) showed a positive correlation with lens transparency but no association with baseline cataract status or 9-year cataract surgery risk [21]. However, cataract surgery may not be an accurate surrogate for cataract development since it is confounded by healthcare access and patient preferences. A cross-sectional study of 161 Chinese ARC patients found no significant association between qPCR-measured LETL and Lens Opacities Classification System III (LOCS III) grading scores. LETL showed moderate negative correlation with Scheimpflug-derived average lens density across different lens regions, particularly in the cortex, but not with maximum density [22]. However, as LETL was measured locally, these findings cannot be extrapolated to systemic telomere length.

To elucidate the relationship between telomere length and cataract development across diverse populations, we analyzed associations between LTL and both the incidence and severity of ARC in two independent cohorts: the UK Biobank, a community-based cohort, and a hospital-based Chinese cohort. Firstly, we examined the longitudinal relationship between LTL and ARC incidence in the UK Biobank. Secondly, we conducted a phenome-wide association study (PheWAS) to validate this association across 1,011 phecodes within the UK Biobank. Finally, we assessed the association between LTL and lens opacities using Scheimpflug imaging in the Chinese cohort.

## Methods

### Study population

#### UK Biobank study

The UK Biobank is a large community-based cohort comprising over 500,000 participants aged 40 to 73 years, recruited from 22 assessment centers across the United Kingdom between 2006 and 2010. Study design details have been published elsewhere [23, 24]. Participants provided comprehensive baseline data on geographic factors, lifestyle, and health-related aspects via questionnaires, interviews, physical measurements, and biological sample collection. For this analysis, we included participants with available LTL data who underwent ophthalmological assessments and had no baseline self-reported eye diseases. The detailed study protocol of UK Biobank is available online (https://www.ukbiobank.ac.uk/).

Ethics approval for the UK Biobank study was obtained from the National Information Governance Board for Health and Social Care and the NHS Northwest Multicenter Research Ethics Committee (11/NW/0382). All participants provided informed consent electronically at the baseline assessment, in accordance with the Declaration of Helsinki. This study was conducted under UK Biobank application number 86091.

#### Chinese cohort

From March to November 2023, 230 cataract patients from the Ophthalmology Clinic of Guangdong Provincial People's Hospital were screened using medical record reviews, structured interviews, and comprehensive ocular examinations. During structured interviews, baseline data on geographic factors, lifestyle, and health-related aspects were collected. Comprehensive ocular examinations included visual acuity testing, intraocular pressure (IOP) measurement, slit-lamp examination and Scheimpflug imaging. Blood samples were obtained for LTL measurement. Exclusion criteria included non-ARC subtypes (traumatic, radiation, pediatric, or secondary cataracts), coexisting ophthalmic conditions (anterior segment disorders or retinal pathologies), history of intraocular surgery [except uncomplicated phacoemulsification and intraocular lens (IOL) implantation], and major systemic comorbidities (cancer, autoimmune, metabolic, or genetic diseases). Patients with history of unilateral cataract surgery were retained, but only their phakic eyes were eligible for Scheimpflug analysis. Additional inclusion criteria were valid LTL measurements and eligible Scheimpflug imaging.

Written informed consent was obtained from all participants. This study was approved by the Medical Research Ethics Committee of Guangdong Provincial People’s Hospital (KY2023-1210–02) and adhered to the principles of the Declaration of Helsinki.

### Telomere length measurement

In both cohorts, blood samples were collected in Vacutainers (EDTA tubes) and stored under appropriate refrigeration. Genomic DNA was extracted from peripheral blood leukocytes, and relative mean LTL was measured using multiplex qPCR to quantify the ratio of telomere amplification product (T) to a single-copy gene (S) [25].

For the UK Biobank, DNA extraction and statistical adjustment protocols were published previously [26]. Despite the project's scale and duration, statistical adjustments minimized technical and inter-assay variation. The technically adjusted LTL was log-transformed for normalization and Z-standardized across all individuals with LTL measurements [27]. In the Chinese cohort, genomic DNA was extracted in a single batch using the TIANamp Blood DNA Kit (TIANGEN, DP348), with LTL measured in a single run using the Absolute Human Telomere Length Quantification qPCR Assay Kit (ScienCell, 8918). All samples were run in duplicate by the same technician and under identical conditions for quality control and checked for agreement between the duplicate values. Samples with high variable values (> 10%) were rerun and reanalyzed [28]. LTL was log-transformed for normalization and Z-standardized.

### Ascertainment of cataract cases

In both cohorts, ARC was identified using the International Classification of Diseases (ICD) codes (ICD-10: H250, H251, H252, H258, H259) and the Office of Population Censuses and Surveys Classification of Interventions and Procedures (OPCS) codes (OPCS-4: C71.2, C75.1). Additionally, ARC was ascertained using ICD-9 (code 3661) and OPCS-3 (code 170, 173, 174) in the UK Biobank. The earliest recorded ICD or OPCS code date served as the onset date of ARC.

In the UK Biobank, baseline exclusion criteria included self-reported cataract at baseline, ICD or OPCS codes corresponding to cataract prior to baseline assessment. Self-reported cataracts at baseline were ascertained if participants selected the corresponding item from a predefined list of answers to the question "Has a doctor ever told you that you have any of the following problems with your eyes?" or stated they ever had cataract surgery. Incident ARC cases during follow-up were ascertained through ICD or OPCS codes recorded after baseline assessment. Follow-up time in the UK Biobank was calculated from the date of baseline assessment and censored at the date of incident cataract events, death, loss to follow-up, or the end of follow-up (April 28, 2021), whichever came first. Person-years were calculated from baseline assessment to the onset date of ARC, death, or the end of follow-up. Variables used in this study from the UK Biobank are detailed in Supplementary Table 1.

### Lens opacities assessment

In the Chinese cohort, cataract severity was objectively measured using Scheimpflug imaging (Pentacam 70,900 HR; Oculus Optikgeräte GmbH, Wetzlar, Germany) in 230 participants following pharmacological dilation. The Scheimpflug camera acquired 25 single-slit images within 2 s using a blue ultraviolet-free light-emitting diode. The software automatically quantified 25 three-dimensional (3D) images of the anterior segment, including lens and corneal densitometry (Supplementary Fig. 1a) [22]. A closed curve was drawn along the lens contour (Supplementary Fig. 1b), and the average and maximum densities of these regions were expressed in pixel intensity units ranging from 0 (transparent lens) to 100 (completely opaque lens).

Pentacam nucleus staging (PNS) tools were used to obtain Pentacam densitometry of zones (PDZ) values, representing 3D zones centered at the corneal apex with diameters of 2 mm (PDZ1), 4 mm (PDZ2), and maximum diameter (PDZM). PDZ height was measured from the anterior to posterior visible lens surfaces (Supplementary Figs. 1c, 1d, and 1e). The software automatically calculated average and maximum densities and graded cataracts based on lens density [29]. The maximal linear density (LDmax), defined as the maximum density on the vertical axis through the corneal apex, was also recorded (Supplementary Fig. 1f). All Scheimpflug images underwent quality screening. Exclusion criteria included eyes with IOL implantation, eyelid/lash obstruction, significant motion artifacts, or media opacity precluding lens visualization. For eligible phakic eyes, three optimal-quality images per eye showing full cross-sectional visibility of the lens nucleus/cortex without specular reflections or misalignment were selected. Mean densitometry values were derived from triplicate measurements. The surgical candidate eye (if meeting quality standards) was prioritized; otherwise, the contralateral phakic eye was analyzed.

### Covariates

Demographic characteristics included age, sex, ethnicity (categorized as White or others), socioeconomic status, and education. Lifestyle factors comprised smoking status, alcohol consumption status, and physical activity levels. Health-related factors included obesity and comorbidities (diabetes, hypertension, and hyperlipidemia). Hypertension was defined as self-reported or physician-diagnosed, taking antihypertensive drugs, or having a systolic blood pressure of at least 130 mmHg or a diastolic blood pressure of at least 80 mmHg averaged over two measurements. Diabetes included self-reported or physician-diagnosed diabetes, medication use, or a glycosylated hemoglobin level of ≥ 6.5%. Hyperlipidemia was defined as physician-diagnosed, medication use, or a total cholesterol level ≥ 6.21 mmol/L [24]. Obesity was defined as body mass index (BMI) ≥ 30 kg/m^2^ in the UK Biobank and ≥ 28 kg/m^2^ in the Chinese cohort [30, 31]. Socioeconomic status was assessed using the Townsend deprivation index in the UK Biobank and self-reported monthly income in the Chinese cohort [32]. In the Chinese cohort focused on severity phenotypes, additional adjustments included cardiovascular disease history, duration of blurred vision, best-corrected visual acuity (BCVA), and IOP [33–36].

### Statistical analysis

Baseline characteristics were reported using descriptive statistics, including means and standard deviations (SD) for continuous variables, and numbers and percentages for categorical variables. Unpaired t-tests were used to compare means between groups for continuous variables, while Pearson χ^2^ tests were used to compare distributions between groups for categorical variables.

In the UK Biobank, Cox proportional hazards models were used to calculate the hazard ratio (HR) and 95% confidence interval (CI) for the association between LTL and cataract risk. The proportional hazards assumption was tested by analyzing the relationship between standardized Schoenfeld residuals and time, with no violations detected. To assess the robustness of the association, two nested Cox models were established. Model 1 was adjusted for age, sex, and ethnicity; Model 2 was additionally adjusted for Townsend deprivation index, education, smoking status, alcohol consumption status, obesity, physical activity levels, history of hypertension, diabetes and hyperlipidemia. Restricted cubic splines with four knots were used to flexibly model and visualize the nonlinear relationship between LTL and ARC incidence. Subgroup analysis was performed by sex and age. Sensitivity analysis was conducted by excluding incident cases in the first two years of follow-up.

For the PheWAS, disease outcome data were obtained through linkage to Hospital Episode Statistics and Mortality Statistics until April 28, 2021. ICD-9/10 codes were used to extract all entries, which were then converted into phenotype codes (phecodes) aligned with diseases commonly used in clinical practice and genomics research [37]. Participants with a phecode were classified as cases; those without phecodes in the same category served as controls. To ensure robust statistical power, phecodes with fewer than 200 cases were excluded, resulting in 1,011 phecodes for analysis [38]. The association between LTL and each phenotype was adjusted for age, sex, BMI, assessment center, and the first ten genetic principal components. Bonferroni correction (*P* < 4.92 × 10⁻^5^) was applied to account for multiple testing. In the Chinese cohort, multivariate linear regression analysis was used to assess the association between LTL and lens opacity indicators from Scheimpflug imaging, adjusting for age, sex, monthly income, education, smoking status, alcohol consumption status, obesity, physical activity levels, history of hypertension, diabetes, hyperlipidemia, cardiovascular disease history, duration of blurred vision, BCVA and IOP.

All analysis were conducted using R (v.4.3.1, R Foundation for Statistical Computing) and Stata (v.17, StataCorp LP). Statistical significance was considered when *P* < 0.05.

## Results

### Demographics and characteristics of UK Biobank

From 502,383 UK Biobank participants, 122,932 individuals (54.8% females) with a mean age of 56.27 ± 8.10 years were included. The median follow-up time was 11.18 years, during which 4,089 participants (33.26%) developed cataracts. Baseline characteristics stratified by incident cataract status are detailed in Supplementary Table 2. Participants who developed cataracts during follow-up were significantly older, more likely to be female, non-White, obese, less educated, and non-drinkers, with higher smoking rates and greater prevalence of diabetes, hypertension, and hyperlipidemia at the baseline assessment (all *P* < 0.001). Characteristics stratified by LTL quantiles are presented in Supplementary Table 3. The mean baseline relative LTL was 0.003 ± 1.000. Participants with shorter LTL tended to be older, male, White, obese, less educated, socioeconomically deprived, smokers, drinkers, and more likely to have diabetes, hypertension, or hyperlipidemia (all *P* < 0.001). Demographic characteristics of UK Biobank participants are summarized in Table 1.

**Table 1:** Baseline characteristics of UK Biobank and Chinese cohort participants

Baseline characteristic	UK Biobank cohort	Chinese cohort
Number of participants	122,932	53
Age, mean (SD, years)	56.27 (8.10)	71.74 (9.66)
Sex, No. (%)		
Female	67,344 (54.78)	33 (62.26)
Male	55,588 (45.22)	20 (37.74)
Ethnicity, No. (%)		
White	112,261 (91.32)	0
Non-white	10,671 (8.68)	53 (100.00)
Townsend index, mean (SD)	− 1.03 (3.04)	NA
Monthly income, mean (SD), CNY	NA	4806.38 (4693.54)
Education, No. (%)		
Others	80,490 (65.48)	40 (75.47)
College or university degree	42,442 (34.52)	13 (24.53)
Smoking status, No. (%)		
Never	67,762 (55.31)	41 (82.00)
Former/current	54,751 (44.69)	9 (18.00)
Drinking status, No. (%)		
Never	5,883 (4.79)	39 (78.00)
Former/current	116,934 (95.21)	1 (22.00)
Obesity, No. (%)		
No	92,670 (75.83)	51 (96.23)
Yes	29,538 (24.17)	2 (3.77)
Physical activity, No. (%)		
Not meeting recommendation	17,939 (17.89)	18 (33.96)
Meeting recommendation	82,335 (82.11)	35 (66.04)
History of diabetes, No. (%)		
No	116,854 (95.06)	53 (100.00)
Yes	6,078 (4.94)	0
History of hypertension, No. (%)		
No	33,316 (27.10)	24 (45.28)
Yes	89,616 (72.90)	29 (54.72)
History of hyperlipidemia, No. (%)		
No	67,126 (54.60)	44 (83.02)
Yes	55,806 (45.40)	9 (16.98)

*SD* = standard deviation; *NA* = not available; *CNY* = Chinese Yuan

### LTL and incidence of ARC

Analysis treating LTL as a continuous variable revealed a significant inverse association with incident ARC after adjusting for age, sex, and ethnicity (HR = 0.93, 95% CI: 0.91 to 0.96; *P* < 0.001). This association persisted in the fully adjusted model (HR = 0.93, 95% CI: 0.90 to 0.97; *P* < 0.001). When stratified by LTL quartiles, participants in the highest quartile had significantly lower ARC risk versus the lowest quartile (Model 2: adjusted HR = 0.84, 95% CI: 0.75 to 0.93; *P* = 0.001). Dose–response relationships were evident in both models (Model 1: *P* for trend < 0.001; Model 2: *P* for trend = 0.001; Table 2). Subgroup analysis by age and sex showed no significant interactions (all *P* for interaction > 0.05; Supplementary Table 4). Results remained robust after excluding incident cases within the first two follow-up years.

**Table 2:** Multivariable cox regression for incident cataract associated with LTL in UK Biobank

LTL		Incident cataract	Model 1		Model 2	
			HR (95% CI)	*P*	HR (95% CI)	*P*
All participants	Mean (SD)	No. of cases/controls				
LTL (continuous variable)						
− 9.422 to 10.603	0.003 (1.00)	4,089/118,843	0.93 (0.91–0.96)	** < 0.001**	0.93 (0.90–0.97)	** < 0.001**
LTL (categorical variable)						
Q1 (− 9.422 to − 0.645)	− 1.251 (0.54)	1,330/29,403	1 [Reference]	NA	1 [Reference]	NA
Q2 (− 0.645 to 0.000)	− 0.308 (0.19)	1,063/29,670	0.89 (0.83–0.97)	**0.007**	0.90 (0.82–0.98)	**0.020**
Q3 (0.000 to 0.649)	0.313 (0.19)	928/29,805	0.87 (0.80–0.95)	**0.002**	0.89 (0.81–0.98)	**0.016**
Q4 (0.649 to 10.603)	1.261 (0.56)	768/29,965	0.83 (0.76–0.91)	** < 0.001**	0.84 (0.75–0.93)	**0.001**
*P* for trend				** < 0.001**		**0.001**
Excluding incident cataract within two years						
LTL (continuous variable)						
− 9.422 to 10.603	0.004 (1.00)	3,664/118,843	0.93 (0.90–0.97)	** < 0.001**	0.93 (0.90–0.97)	** < 0.001**
LTL (categorical variable)						
Q1 (− 9.422 to − 0.645)	− 1.251 (0.54)	1,194/29,403	1 [Reference]	NA	1 [Reference]	NA
Q2 (− 0.645 to 0.000)	− 0.308 (0.19)	945/29,670	0.88 (0.81–0.96)	**0.005**	0.89 (0.80–0.98)	**0.014**
Q3 (0.000 to 0.649)	0.313 (0.19)	834/29,805	0.87 (0.80–0.95)	**0.003**	0.89 (0.80–0.98)	**0.023**
Q4 (0.649 to 10.603)	1.261 (0.56)	691/29,965	0.83 (0.76–0.91)	** < 0.001**	0.83 (0.75–0.93)	**0.001**
*P* for trend				** < 0.001**		**0.001**

Model 1 has been adjusted for age, sex and ethnicity. Model 2 has been adjusted for age, sex, ethnicity, Townsend index, education, smoking status, alcohol consumption status, obesity, physical activity levels, history of hypertension, diabetes, hyperlipidemia. Bold values denote statistical significance at the *P* < 0.05 level

*LTL* = leukocyte telomere length; *SD* = standard deviation; *HR* = hazard ratio; *CI* = confidence interval; *Q* = quartile; *NA* = not available

Restricted cubic spline analysis revealed a nonlinear L-shaped association in both models (*P* for nonlinearity = 0.03). Cataract risk decreased steeply with longer LTL until a threshold, beyond which it plateaued. This pattern was consistent in sex-stratified analysis (Supplementary Fig. 2).

PheWAS confirmed the telomere-cataract association at the phenome-wide significance level (Bonferroni-corrected *P* < 4.92 × 10⁻^5^; Supplementary Fig. 3). Among 1,011 outcomes, cataract was significantly associated with telomere length (odds ratio = 0.97, 95% CI: 0.96 to 0.98; *P* = 2.36 × 10⁻⁶).

### LTL and severity of ARC

**Fig. 1:**
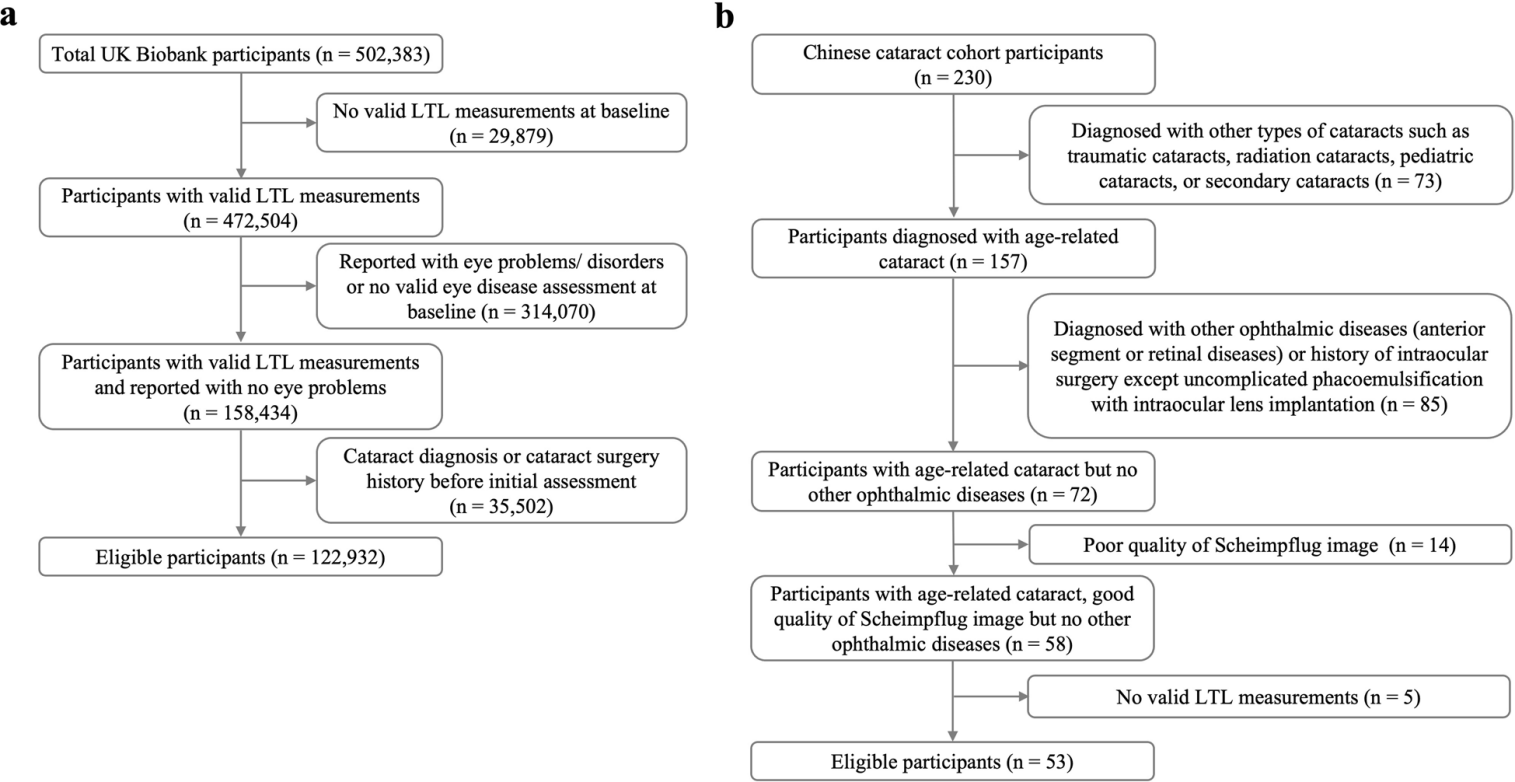
Flowchart of participants included in the two cohorts.** a** Flowchart of participants recruited from the UK Biobank, a community-based cohort. **b** Flowchart of participants recruited from a Chinese hospital-based cohort. LTL, leukocyte telomere length

After applying the exclusion criteria, a final number of 53 participants (mean age 71.74 ± 9.66 years; 62.3% female) were enrolled for analysis. From 106 initially eligible eyes, quality assessment excluded 10 pseudophakic eyes (IOL-implanted: OD = 7, OS = 3) and 21 eyes with poor-quality images/acquisition failure (OD = 15, OS = 6). Thus, 75 eyes (OD = 31, OS = 44) from 53 participants qualified for the Pentacam analysis. Mean baseline relative LTL was 0.01 ± 1.03, with average blurred vision duration of 21.68 ± 23.29 months. The study inclusion flowcharts of the two cohorts are shown in Fig. 1. Demographic characteristics of participants at baseline for both cohorts are summarized in Table 1. Lens opacity indicators measured by Scheimpflug imaging in the Chinese cohort are provided in Table 3.

**Table 3:** Measurements of lens densities using Scheimpflug imaging in the Chinese cohort

Variables	OD (n = 31)		OS (n = 44)	
	Mean (SD)	Median	Mean (SD)	Median
Average whole lens density	9.37 (1.21)	9.00	9.65 (1.06)	9.40
Maximum whole lens density	52.41 (24.20)	42.87	55.45 (24.75)	48.05
Average PDZ1 density	11.29 (1.42)	11.05	11.40 (1.46)	11.10
Average PDZ2 density	10.65 (1.05)	10.60	10.87 (1.02)	10.70
Average PDZM density	10.44 (0.96)	10.65	10.62 (0.80)	10.70
Pentacam nucleus staging	1.00 (0.59)	1.00	1.11 (0.39)	1.00
Maximal linear density	37.95 (20.00)	32.73	39.27 (20.54)	32.22

*OD* = oculus dexter; *OS* = oculus sinister; *PDZ* = Pentacam densitometry of zones; *SD* = standard deviation

Multivariate analysis revealed significant inverse associations between LTL and lens opacity indicators from Scheimpflug imaging, including PDZ2 (β = − 0.45, 95% CI: − 0.83 to − 0.07; *P* = 0.02) and average whole lens density (β = − 0.32, 95% CI: − 0.61 to − 0.04; *P* = 0.03) (Supplementary Fig. 4). Other lens opacity indicators showed no significant relationship with LTL.

## Discussion

This study identified a dose–response inverse association between LTL and incident ARC, characterized by a distinct L-shaped relationship in community-based participants. PheWAS confirmed that cataract was significantly associated with telomere length at the phenome-wide significance level. Additionally, we observed a significant inverse relationship between LTL and cataract severity in hospital-based participants. These complementary findings from two independent cohorts collectively supported an inverse LTL-cataract relationship. Our findings aligned with the LensAge index, a deep learning-based biological age from lens photographs, which integrates lenticular and systemic aging [39].

Oxidative stress, which is implicated in both cataract pathogenesis and telomere attrition, may explain the LTL-cataract association [40, 41]. Telomere length captures biological variability independent of chronological age and is highly susceptible to reactive oxygen species (ROS)-induced damage in vitro [41, 42]. Oxidative stress depletes telomeric repeat-binding factors (TRF1/TRF2), which are essential for telomere replication and T-loop formation. Moreover, oxidative stress-induced 8-oxo-7,8-dihydroguanine (8-oxoG) causes replication fork stalling at telomeres, driving telomere dysfunction and cellular senescence [43]. ARC patients exhibited increased lipid peroxidation products in aqueous humor, higher proportions of LECs with DNA breaks and shorter LETL [44, 45]. These findings implicated oxidative DNA damage and telomere attrition in cataract development.

Notably, our analysis revealed that the LTL-lens opacity association was strongest in nuclear regions. Lens fibers derive from epithelial cells that migrate toward the nucleus, losing mitochondria and diminishing antioxidant capacity [46]. Consequently, the lens nucleus, which is composed of older fibers, accumulates lifelong oxidative damage and increases opacity over decades [47].

The strengths of this study include its large-scale prospective design with long-term follow-up, rigorous adjustment for multidimensional confounders, and the use of multiple analytical approaches, such as dose–response modeling and restricted cubic splines. The cross-cohort design, incorporating data from both UK community-dwelling and Chinese hospital-based populations, adds robustness to our conclusions. However, several limitations warrant consideration. First, fundamental methodological differences between cohorts precluded external validation. The hospital-based Chinese cohort may lack generalizability to community-dwelling elders, while the UK Biobank's ‘healthy volunteer’ bias remains a concern. Second, algorithmically defined cataract outcomes in the UK Biobank predominantly relied on hospital records. This approach has likely missed mild or subclinical cases. Third, due to the lack of cataract subtype data in the UK Biobank, we could not examine subtype-specific LTL associations. Given established etiological differences among subtypes, future studies should implement granular phenotyping [45, 47, 48]. Fourth, substantial sample size disparity of the two cohorts affects the robustness and generalizability of our severity-related findings. Future multiethnic cohorts with balanced designs should replicate these findings. Lastly, while statistically robust, the modest effect size (HR = 0.93) precludes clinical utility for individual prediction. Instead, these findings highlight the lens, constantly exposed to pro-aging stressors, as a sentinel tissue revealing systemic oxidative burden and telomere dynamics.

Our cross-cohort analysis reveals a dose–response association between longer LTL and reduced cataract risk and severity, highlighting a potential mechanistic link between lenticular and systemic biological aging. LTL reflects cumulative oxidative/inflammatory burden from environmental and lifestyle factors, a composite index summarizing lifelong stress exposure relevant to cataractogenesis. Non-regenerative nature of the lens magnifies systemic aging signatures. Our findings demonstrate that modifiable lifestyle factors reducing oxidative burden may concurrently preserve LTL and delay cataractogenesis and extend relevance to aging intervention research beyond ophthalmology.

## Conclusions

In conclusion, our study demonstrates that longer LTL is associated with a reduced risk and severity of ARC, suggesting shared biological pathways between lenticular and systemic aging. These findings support the lens as a sentinel tissue for systemic aging and LTL as an index of lifelong stress exposure relevant to cataractogenesis. Moreover, modifiable factors that reduce oxidative burden may concurrently preserve telomere length and delay cataract formation.

## Supplementary Information

Additional file1 (DOCX 2106 kb)

## Data Availability

The datasets used and analyzed during the current study are available from the corresponding author on reasonable request.

## Abbreviations

LTL: Leukocyte telomere length
ARC: Age-related cataract
LETL: Lens epithelium telomere length
LEC: Lens epithelial cell
qPCR: Quantitative polymerase chain reaction
FISH: Fluorescence in situ hybridization
OPCS: Office of Population Censuses and Surveys Classification of Interventions and Procedures
ICD: International Classification of Diseases
LOCS III: Lens Opacities Classification System III
IOL: Intraocular lens
PDZ: Pentacam densitometry of zones
PNS: Pentacam nucleus staging
LDmax: Maximal linear density
PheWAS: Phenome-wide association study
BCVA: Best-corrected visual acuity
IOP: Intraocular pressure
BMI: Body mass index
OD: Oculus dexter
OS: Oculus sinister
