# Supplementary material for: Impacts of leukocyte telomere length on incidence and severity of age-related cataract: a cross-cohort analysis
Source: Eye Vis (Lond). 2025 Dec 1;12:50. doi: 10.1186/s40662-025-00465-x (PMC12667178; doi:10.1186/s40662-025-00465-x)

**Supplementary Material**

**Supplementary Table 1.** UK biobank showcase variables used in the paper.

| **Measurements** | **Field ID** | **Time** | **Description** |
| --- | --- | --- | --- |
| **Visual health** | | | |
| Self-reported non-cancer illness | 20002 | Baseline assessment | The procedure for verbal interview at an Assessment Center of the UK Biobank follow the manual: https:// biobank.ndph.ox.ac.uk/ukb/ukb/docs/Interview.pdf |
| Age cataract diagnosed | 4700 | Baseline assessment | Touchscreen question "What was your age when a cataract was first diagnosed?" |
| Ever had cataract surgery | 5324 | Baseline assessment | If the participant stated they had eye surgery (Field 5181), they were asked if they had cataract surgery |
| Self-reported eye disorders  (including cataract, glaucoma, macular degeneration, and diabetes-related eye disease) | 6148 | Baseline assessment | Touchscreen question "Has a doctor told you that you have any of the following problems with your eyes? (You can select more than one answer)" |
| **Age-related cataract** | | | |
| Incident cataract | 41270 (code H250, H251, H252, H258, H259) | From the date of baseline assessment to the date of cataract ascertainment | Hospital inpatient records with cataract as main or any secondary diagnoses based on the 10th edition of the WHO International Classification of Diseases (ICD-10) |
|  | 41271 (code 3661) |  | Hospital inpatient records with cataract as main or any secondary diagnoses based on the 9th edition of the WHO International Classification of Diseases (ICD-9) |
|  | 41272 (code C71.2, C75.1) |  | Hospital inpatient records with cataract surgery in either the main or secondary position based on the Office of Population Censuses and Surveys Classification of Interventions and Procedures, version 4 (OPCS-4) |
|  | 41273 (code170, 173, 174) |  | Hospital inpatient records with cataract surgery in either the main or secondary position based on the Office of Population Censuses and Surveys Classification of Interventions and Procedures, version 3 (OPCS-3) |
| **Leucocyte telomere length** | | | |
| Leucocyte telomere length | 22192 | Baseline assessment | Technically adjusted leucocyte telomere length (Field 22191) which has been both log-transformed to obtain a normal distribution and then Z-standardized using the distribution of all individuals with a telomere length measurement |
| **Demographic information** | | | |
| Age | 21003 | Baseline assessment | Refer to the age of the participant on the day they attended an Assessment Center |
| Sex | 31 | Baseline assessment | Sex of participant |
| Ethnic background | 21000 | Baseline assessment | Recorded as white or non-white |
| Townsend deprivation index | 189 | Baseline assessment | Townsend deprivation index calculated immediately prior to participant joining UK Biobank based on the preceding national census output areas |
| Education | 6138 | Baseline assessment | Touchscreen question "Which of the following qualifications do you have? (You can select more than one)" |
| Physical activity levels | 22036 | Baseline assessment | Indicates whether a person met the 2017 UK Physical activity guidelines of 150 minutes of walking or moderate activity per week or 75 minutes of vigorous activity |
| **Risk factors** | | | |
| Hypertension | 20002 (code 1065, 1072) | Baseline assessment | Self-reported hypertension |
|  | 6153  (code 2) |  | Use of antihypertensive drugs |
|  | 4080 |  | Average systolic blood pressure of at least 130 mmHg |
|  | 4079 |  | Average diastolic blood pressure of at least 80 mmHg |
| Diabetes mellitus | 2443 | Baseline assessment | Doctor-diagnosed diabetes mellitus. Touchscreen question "Has a doctor ever told you that you have diabetes?" |
|  | 20003 |  | Use of anti-hyperglycemic medications |
|  | 6153  (code 3) |  | Use of insulin |
|  | 30750 |  | Glycated hemoglobin level measured by HPLC analysis on a Bio-Rad VARIANT II Turbo (≥ 48 mmol/mol) |
| Hyperlipidemia | 20002  (code 1473) | Baseline assessment | Self-reported hyperlipidemia |
|  | 6153 |  | Use of statins |
|  | 20003 |  | Use of hyperlipidemia-related medication |
|  | 30690 |  | Blood cholesterol level Measured by CHO-POD analysis on a Beckman Coulter AU5800 (≥ 6.21 mmol/L) |
| Smoking status | 20116 | Baseline assessment | This field summarizes the current/past smoking status of the participant |
| Alcohol consumption | 20117 | Baseline assessment | This field summarizes the current/past alcohol drinker status of the participant |
| Obesity | 21001 | Baseline assessment | Body mass index (BMI) value is constructed from height and weight. Obesity was defined as BMI ≥ 30 kg/m^2^ |

HPLC = high-performance liquid chromatography; CHO-POD = cholesterol oxidase-peroxidase.

**Supplementary Table 2.** Demographic characteristics of study participants at baseline examination and stratified by incident cataract of the UK Biobank cohort.

| **Baseline characteristic** | **Total** | **Mean (SD)/N (%)** | | ***P*** |
| --- | --- | --- | --- | --- |
|  |  | **Incident cataract** | **Control** |  |
| Number of participants | 122,932 | 4,089 (33.26) | 118,843 (96.67) |  |
| Age, mean (SD, years) | 56.27 (8.10) | 62.56 (5.59) | 56.05 (8.08) | **< 0.001** |
| Sex, No. (%) |  |  |  | **< 0.001** |
| Female | 67,344 (54.78) | 2,368 (57.91) | 64,976 (54.67) |  |
| Male | 55,588 (45.22) | 1,721 (42.09) | 53,867 (45.33) |  |
| Ethnicity, No. (%) |  |  |  | **< 0.001** |
| White | 112,261 (91.32) | 3,661 (89.53) | 108,600 (91.38) |  |
| Non-white | 10,671 (8.68) | 428 (10.47) | 10,243 (8.62) |  |
| Townsend index, mean (SD) | −1.03 (3.04) | −0.97 (3.11) | −1.03 (3.10) | 0.1626 |
| Education, No. (%) |  |  |  | **< 0.001** |
| Others | 80,490 (65.48) | 2,948 (72.10) | 77,542 (65.25) |  |
| College/University | 42,442 (34.52) | 1,141 (27.90) | 41,301 (34.75) |  |
| Smoking status, No. (%) |  |  |  | **< 0.001** |
| Never | 67,762 (55.31) | 2,068 (50.82) | 65,694 (55.46) |  |
| Former/current | 54,751 (44.69) | 2,001 (49.18) | 52,750 (44.54) |  |
| Drinking status, No. (%) |  |  |  | **< 0.001** |
| Never | 5,883 (4.79) | 28 (6.86) | 5,603 (4.72) |  |
| Former/current | 116,934 (95.21) | 3,803 (93.14) | 113,131 (95.28) |  |
| Obesity, No. (%) |  |  |  | **< 0.001** |
| No | 92,670 (75.83) | 2,887 (71.09) | 89,783 (75.99) |  |
| Yes | 29,538 (24.17) | 1,174 (28.91) | 28,364 (24.01) |  |
| Physical activity, No. (%) | | |  | 0.518 |
| Not meeting recommendation | 17,939 (17.89) | 589 (18.32) | 17,350 (17.88) |  |
| Meeting recommendation | 82,335 (82.11) | 2,626 (81.68) | 79,709 (82.12) |  |
| History of diabetes, No. (%) |  |  |  | **< 0.001** |
| No | 116,854 (95.06) | 3,695 (90.36) | 113,159 (95.22) |  |
| Yes | 6,078 (4.94) | 394 (9.64) | 5,684 (4.78) |  |
| History of hypertension, No. (%) |  |  |  | **< 0.001** |
| No | 33,316 (27.10) | 750 (18.34) | 32,566 (27.40) |  |
| Yes | 89,616 (72.90) | 3,339 (81.66) | 86,277 (72.60) |  |
| History of hyperlipidemia, No. (%) |  |  |  | **< 0.001** |
| No | 67,126 (54.60) | 1,693 (41.40) | 65,433 (55.06) |  |
| Yes | 55,806 (45.40) | 2,396 (58.60) | 53,410 (44.94) |  |

SD = standard deviation. Bold values denote statistical significance at the *P* < 0.05 level.

**Supplementary Table 3.** Demographic characteristics of study participants at baseline examination and stratified by quantiles of LTL of the UK Biobank cohort.

| **Baseline characteristic** | **Total** | | | | **LTL** | | | | ***P*** |
| --- | --- | --- | --- | --- | --- | --- | --- | --- | --- |
|  |  |  |  |  | **Q1** | **Q2** | **Q3** | **Q4** |  |
| Number of participants | 122,932 | | | | 30,733 | 30,733 | 30,733 | 30,733 |  |
| Age, mean (SD, years) | 56.27 (8.10) | | | | 58.37 (7.63) | 56.95 (7.90) | 55.66 (8.09) | 54.09 (8.13) | **< 0.001** |
| Sex, No. (%) |  | | | |  |  |  |  | **< 0.001** |
| Female | 67,344 (54.78) | | | | 15,004 (48.82) | 16,286 (52.99) | 17,294 (56.27) | 18,760 (61.04) |  |
| Male | 55,588 (45.22) | | | | 15,729 (51.18) | 14,447 (47.01) | 13,439 (43.73) | 11,973 (38.96) |  |
| Ethnicity, No. (%) |  | | | |  |  |  |  | **< 0.001** |
| White | 112,261 (91.32) | | | | 28,856 (93.89) | 28,495 (92.72) | 28,000 (91.11) | 26,910 (87.56) |  |
| Non-white | 10,671 (8.68) | | | | 1,877 (6.11) | 2,238 (7.28) | 2,733 (8.89) | 3,823 (12.44) |  |
| Townsend index, mean (SD) | −1.03 (3.04) | | | | −1.17 (3.00) | −1.08 (3.03) | −1.02 (3.03) | −0.86 (3.10) | **< 0.001** |
| Education, No. (%) |  | | |  | | | | | **< 0.001** |
| College or university degree | 42,442 (34.52) | | | | 9,617 (31.29) | 10,201 (33.19) | 10,869 (35.37) | 11,755 (38.25) |  |
| Others | 80,490 (65.48) | | | | 21,116 (68.71) | 20,532 (66.81) | 19,864 (64.63) | 18,978 (61.75) |  |
| Smoking status, No. (%) |  | | | |  |  |  |  | **< 0.001** |
| Never | 67,762 (55.31) | | | | 15,941 (52.06) | 16,732 (54.64) | 17,168 (56.06) | 17,921 (58.49) |  |
| Former/current | 54,751 (44.69) | | | | 14,682 (47.94) | 13,892 (45.36) | 13,458 (43.94) | 12,719 (41.51) |  |
| Drinking status, No. (%) |  | | | |  |  |  |  | **< 0.001** |
| Never | 5,883 (4.79) | | | | 1,338 (4.36) | 1,386 (4.51) | 1,481 (4.82) | 1,678 (5.47) |  |
| Former/current | 116,934 (95.21) | | | | 29,366 (95.64) | 29,313 (95.49) | 29,231 (95.18) | 29,024 (94.53) |  |
| Obesity, No. (%) |  | | | |  |  |  |  | **< 0.001** |
| No | 92,670 (75.83) | | | | 22,742 (74.39) | 23,099 (75.58) | 23,225 (76.04) | 23,604 (77.31) |  |
| Yes | 29,538 (24.17) | | | | 7,831 (25.61) | 7,462 (24.42) | 7,319 (23.96) | 6,926 (22.69) |  |
| Physical activity, No. (%) |  | |  | | |  |  |  | 0.766 |
| Not meeting recommendation | 17,939 (17.89) | | | | 4,422 (17.73) | 4,520 (18.09) | 4,479 (17.83) | 4,518 (17.91) |  |
| Meeting recommendation | 82,335 (82.11) | | | | 20,514 (82.27) | 20,469 (81.91) | 20,639 (82.17) | 20,713 (82.09) |  |
| History of diabetes, No. (%) |  | | |  | | | | | **< 0.001** |
| No | 116,854 (95.06) | | | | 28,950 (94.20) | 29,189 (94.98) | 29,310 (95.37) | 29,405 (95.68) |  |
| Yes | 6,078 (4.94) | | | | 1,783 (5.80) | 1,544 (5.02) | 1,423 (4.63) | 1,328 (4.32) |  |
| History of hypertension, No. (%) |  | | | | |  |  |  | **< 0.001** |
| No | 33,316 (27.10) | | | | 7,574 (24.64) | 7,935 (25.82) | 8,481 (27.60) | 9,326 (30.35) |  |
| Yes | 89,616 (72.90) | | | | 23,159 (75.36) | 22,798 (74.18) | 22,252 (72.40) | 21,407 (69.65) |  |
| History of hyperlipidemia, No. (%) |  |  | | | |  |  |  | **< 0.001** |
| No | 67,126 (54.60) | | | | 15,817 (51.47) | 16,426 (53.45) | 16,991 (55.29) | 17,892 (58.22) |  |
| Yes | 55,806 (45.40) | | | | 14,916 (48.53) | 14,307 (46.55) | 13,742 (44.71) | 12,841 (41.78) |  |

LTL = leukocyte telomere length; SD = standard deviation; Q = quartile. Bold values denote statistical significance at the *P* < 0.05 level.

**Supplementary Table 4.** Subgroup analysis by age or sex for incident cataract associated with LTL in the UK Biobank cohort.

| **LTL (continuous variable)** | | **Incident**  **cataract** | **Model 1** | | | **Model 2** | | |
| --- | --- | --- | --- | --- | --- | --- | --- | --- |
|  |  |  | **HR (95% CI)** | ***P*** | ***P* for interaction** | **HR (95% CI)** | ***P*** | ***P* for interaction** |
| All participants | Mean (SD) | N cases/controls |  |  |  |  |  |  |
| Male | −0.097 (1.00) | 1,721/ 53,867 | 0.93 (0.89–0.98) | **0.004** | 0.750 | 0.93 (0.88–0.98) | **0.004** | 0.936 |
| Female | 0.087 (1.00) | 2,368/ 64,976 | 0.94 (0.90–0.98) | **0.002** |  | 0.94 (0.89–0.98) | **0.009** |  |
| < 57 years | 0.185 (0.988) | 548/ 56,947 | 0.89 (0.82–0.97) | **0.011** | 0.115 | 0.89 (0.81–0.98) | **0.015** | 0.152 |
| ≥ 57 years | −0.157 (0.99) | 3,541/ 61,896 | 0.94 (0.91–0.97) | **< 0.001** |  | 0.94 (0.90–0.98) | **0.001** |  |

Model 1 has been adjusted for age, sex and ethnicity. Model 2 has been adjusted for age, sex, ethnicity, Townsend index, education, smoking status, alcohol consumption status, obesity, physical activity levels, history of hypertension, diabetes, hyperlipidemia. Age stratification at 57 years was based on the median baseline age of the 122,932 UK Biobank participants included in this analysis. Bold values denote statistical significance at the *P* < 0.05 level.

LTL = leukocyte telomere length; SD = standard deviation; HR = hazard ratio; CI = confidence interval.

**Supplementary Figure 1. Scheimpflug images of the lens in the Chinese cohort. a** Overview of 25 three-dimensional (3D) Scheimpflug images. **b** Whole lens density measurement (circled region). **c** PDZ1 density measurement (circled region). **d** PDZ2 density measurement (circled region). **e** PDZM density measurement (circled region). **f** LDmax measurement (peak of the wave). PDZ, Pentacam densitometry of zones; LDmax, maximal linear density.
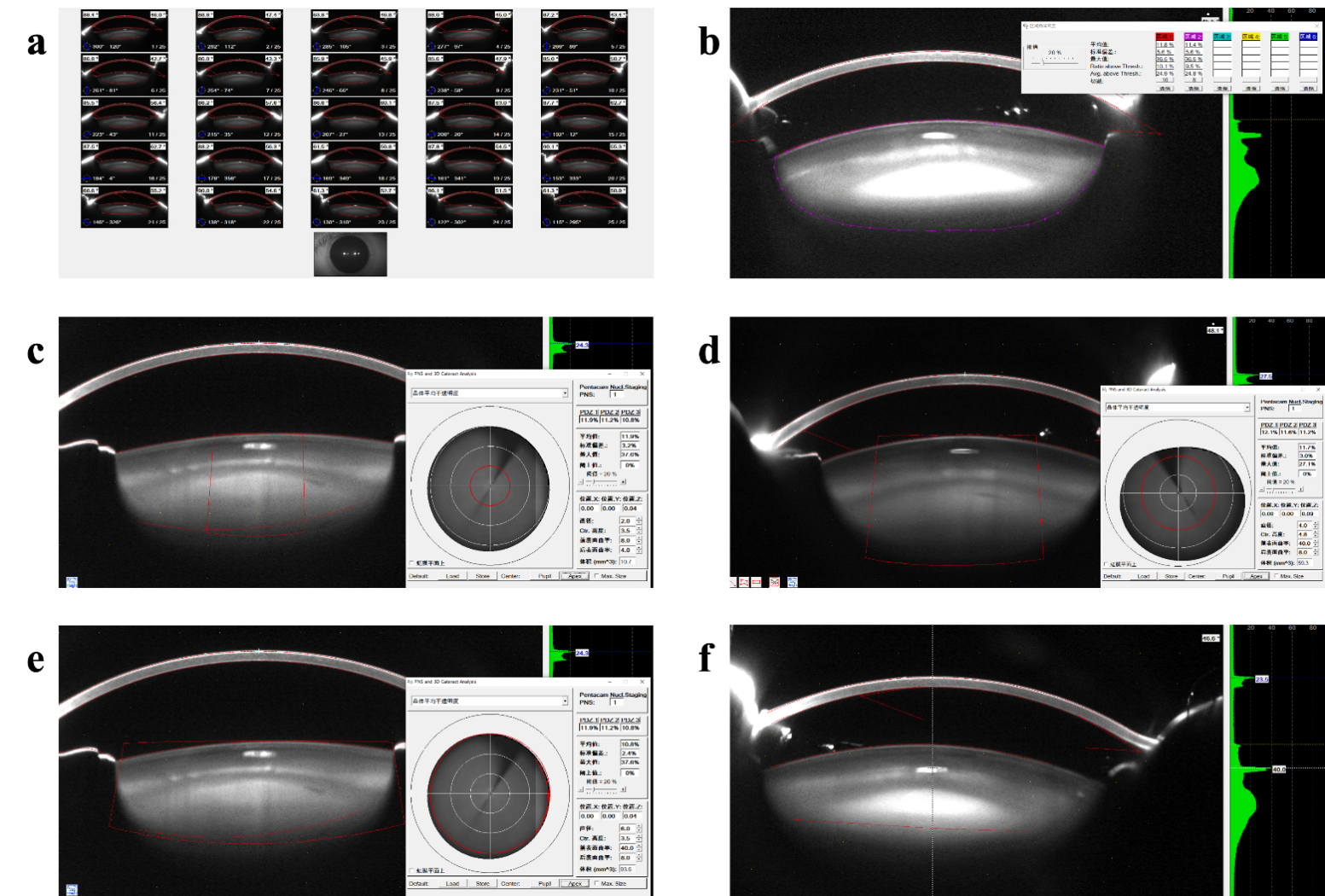


**Supplementary Figure 2. Association between LTL and ARC incidence among UK Biobank participants. a** Adjusted for covariables in model 1. **b** Adjusted for covariables in model 2. **c** Adjusted for covariables in model 1 and stratified by sex. **d** Adjusted for covariables in model 2 and stratified by sex. LTL, leukocyte telomere length; ARC, age-related cataract.


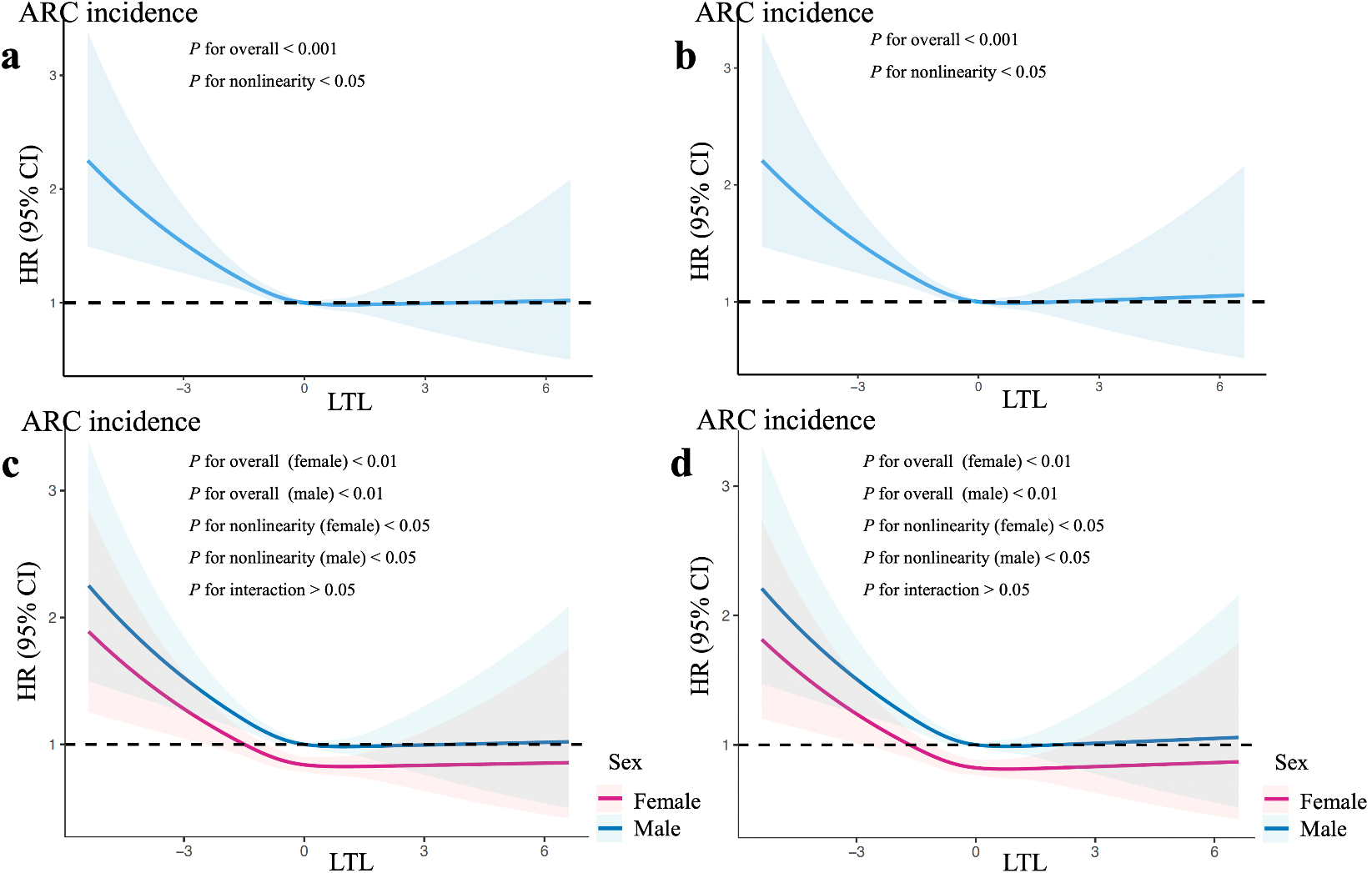


**Supplementary Figure 3. Association between LTL and 1,011 phecodes in the phenome-wide association study.** X-axis corresponds to the list of labels of disease categories; Y-axis corresponds to the minus log-transformed *P* value derived from the phenome-wide association analysis. The red lines indicate the Bonferroni corrected threshold (*P* < 4.92 × 10⁻⁵). LTL, leukocyte telomere length.


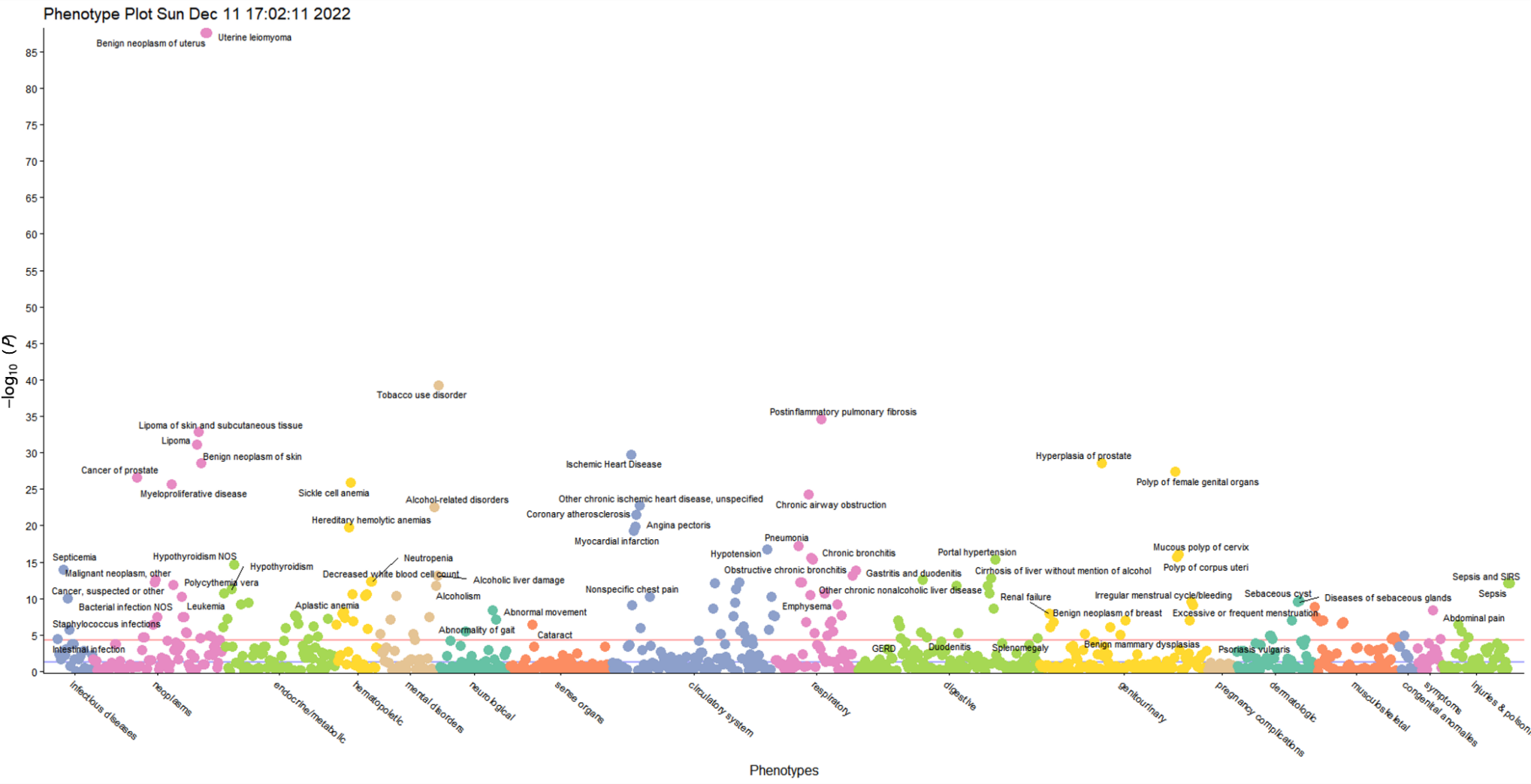


**Supplementary Figure 4. Association between LTL and lens opacity indicators from Scheimpflug imaging.** The multivariate linear regression is adjusted for covariables including age, sex, body mass index (BMI), monthly income, education, smoking status, alcohol consumption status, physical activity levels, history of hypertension, diabetes, hyperlipidemia, cardiovascular disease, duration of blurred vision, best-corrected visual acuity (BCVA), intraocular pressure (IOP). **a** Scatter plot with multivariate linear regression line shows the relationship of LTL (x-axis) with average whole lens density (y-axis). **b** Scatter plot with multivariate linear regression line shows the relationship of LTL (x-axis) with PDZ2 (y-axis). Solid lines indicate regression estimates, shaded area represents 95% CI. LTL, leukocyte telomere length; PDZ, Pentacam densitometry of zones.


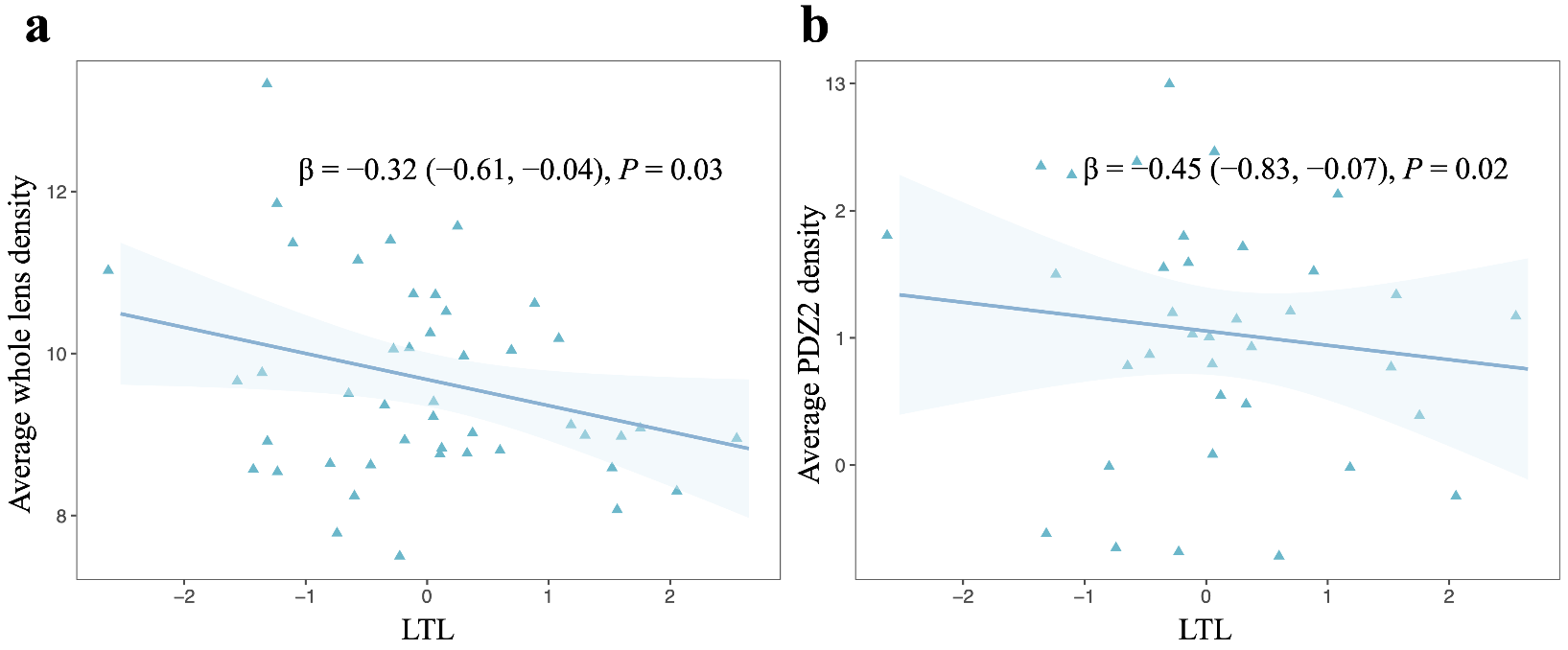

Supplement: Supplementary file 1 — Additional file1 (DOCX 2106 kb) [file 40662_2025_465_MOESM1_ESM.docx]
